# Remote biomonitoring of temperatures in mothers and newborns: design, development and testing of a wearable sensor device in a tertiary-care hospital in southern India

**DOI:** 10.1136/bmjinnov-2016-000153

**Published:** 2018-02-14

**Authors:** Prem K Mony, Prashanth Thankachan, Swarnarekha Bhat, Suman Rao, Maryann Washington, Sumi Antony, Annamma Thomas, Sheela C Nagarajarao, Hiteshwar Rao, Bharadwaj Amrutur

**Affiliations:** 1 Division of Epidemiology & Population Health, St John’s Research Institute, St John’s National Academy of Health Sciences, Bangalore, Karnataka, India; 2 Department of Neonatology, St John’s Medical College Hospital, St John’s National Academy of Health Sciences, Bangalore, Karnataka, India; 3 Department of Obstetrics, St John’s Medical College Hospital, St John’s National Academy of Health Sciences, Bangalore, Karnataka, India; 4 Robert Bosch Center for Cyber Physical Systems, Indian Institute of Science, Bangalore, Karnataka, India; 5 Department of Electrical Communication Engineering, Indian Institute of Science, Bangalore, Karnataka, India

**Keywords:** mother, newborn, remote monitoring, vital signs, wireless sensors

## Abstract

**Objective:**

Newer technologies such as wearables, sensors, mobile telephony and computing offer opportunities to monitor vital physiological parameters and tackle healthcare problems, thereby improving access and quality of care. We describe the design, development and testing of a wearable sensor device for remote biomonitoring of body temperatures in mothers and newborns in southern India.

**Methods:**

Based on client needs and technological requirements, a wearable sensor device was designed and developed using principles of ‘social innovation’ design. The device underwent multiple iterations in product design and engineering based on user feedback, and then following preclinical testing, a techno-feasibility study and clinical trial were undertaken in a tertiary-care teaching hospital in Bangalore, India. Clinical trial phases I and IIa for evaluation of safety and efficacy were undertaken in the following sequence: 7 healthy adult volunteers; 18 healthy mothers; 3 healthy babies; 10 stable babies in the neonatal care intensive unit and 1 baby with morbidities. Time-stamped skin temperature readings obtained at 5 min intervals over a 1-hour period from the device secured on upper arms of mothers and abdomen of neonates were compared against readings from thermometers used routinely in clinical practice.

**Results:**

Devices were comfortably secured on to adults and neonates, and data were efficiently transmitted via the gateway device for secure storage and retrieval for analysis. The mean skin temperatures in mothers were lower than the axillary temperatures by 2°C; and in newborns, there was a precision of –0.5°C relative to axillary measurements. While occasional minimal adverse events were noted in healthy volunteers, no adverse events were noted in mothers or neonates.

**Conclusions:**

This proof-of-concept study shows that this device is promising in terms of feasibility, safety and accuracy (with appropriate calibration) with potential for further refinements in device accuracy and pursuit of further phases of clinical research for improved maternal and neonatal health.

## Introduction

The new sustainable development agenda aims to ensure that the momentum generated by the millennium development goals is continued beyond 2015. India has achieved success in maternal health but is off-track on child health. More vigorous efforts will be needed to meet the new global targets of zero preventable child deaths and a much sharper reduction in maternal deaths by 2030.^1^ Innovation will be central to achieving these goals in maternal and child health; it could be a product, process or healthcare system innovation.^2^ While the unmet health burden will definitely need action on known determinants such as shortage/maldistribution of health workers, coverage of health services, quality of care (QoC) and better monitoring of programmes, ‘disruptive innovations’ in healthcare will also be critical, especially for those at the bottom of the pyramid and traditionally left out of the healthcare net owing to political, economic or geographic reasons.^3^

The disease burden among mothers and neonates is mostly caused by a handful of avoidable conditions—haemorrhage, gestational hypertension, infections and obstructed labour in mothers^4^; and prematurity/low birth weight, infections and birth  asphyxia/birth trauma in newborn babies.^5^ They occur mostly around the time of childbirth and within the first month following delivery. Though the direction of causality is unclear, neonatal hypothermia is associated with all of these three conditions. The prevalence of hypothermia has been found to range from 11% to 92%, and case fatality rates for newborn hypothermia are seen to vary from 9% to 52%.^6^ Currently, in most parts of the world, newborn temperature measurement and documentation are incomplete, resulting in an incomplete understanding of the epidemiology of hypothermia and its clinical consequences.^6^ Remote, real-time monitoring of key physiological parameters,^7^ such as temperature of mothers and newborns, is a promising pathway for the early detection of complications presenting as hypothermia or as fever in mothers/newborns, offering a potential opportunity to impact access as well as QoC for these vulnerable populations. In the development of health technology for surveillance, ‘frugal innovation’ using social innovation design principles such as safety and efficacy; robustness; independence of electricity mains or replaceable batteries; operational capability without needing replacement consumables and simplicity of operation provides an opportunity for the design and deployment of healthcare systems that address access and quality concerns.^8^ Here we describe the design, development and testing of a wearable sensor device for remote biomonitoring (RBM) of body temperatures in mothers and newborns in southern India.

## Methods

### Study setting

St John’s Medical College Hospital, Bangalore, is a 1300-bedded, tertiary-care hospital with 2500 deliveries per annum. It has a Level 3 nursery and takes care of 1000 neonates (inborn:outborn ratio=2:1) in the neonatal intensive care unit (NICU) per year, with survival rates of 99% at 48 hours. About one-third of newborns are low birth weight and one-fifth are preterm. Background rates of maternal mortality ratio and infant mortality ratio were 1.33 and 31 per 1000 live-births for Karnataka state.^9^

### Choice of site of temperature measurement in adult and newborn

**Figure 1:**
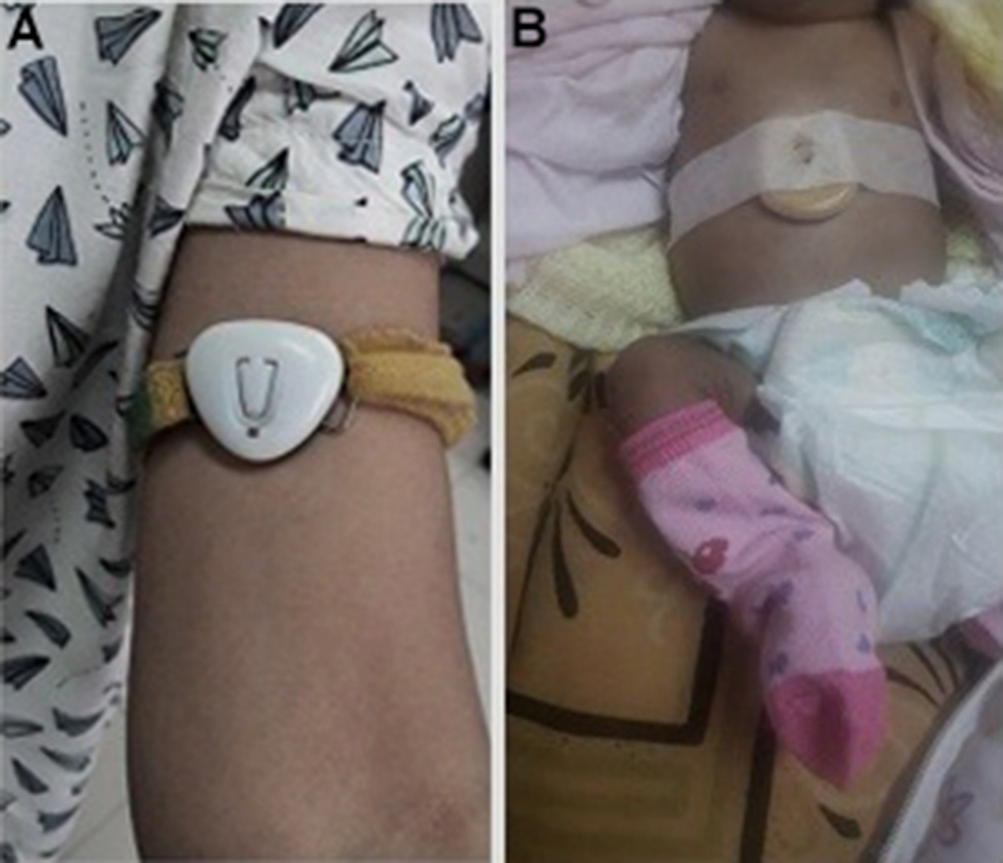
Positioning of the wearable remote biomonitoring sensor for temperature surveillance in (A) mother (upper arm) and (B) newborn (epigastrium).

**Figure 2:**
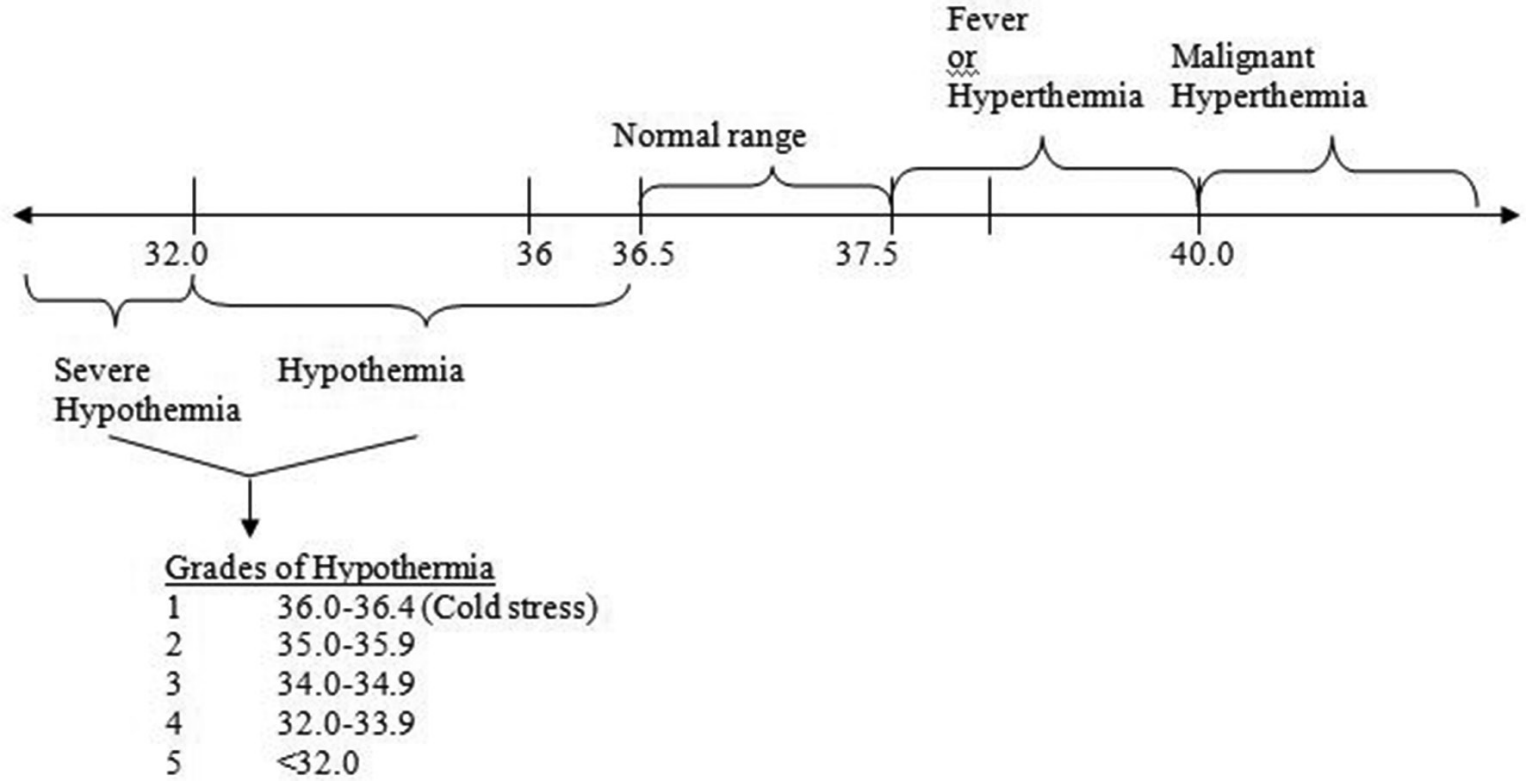
Temperature cut-off for neonates (with alerts beyond the normal range of 36.5–37.5°C).

In the adult, the upper arm was selected for continuous monitoring. While the axillary or other sites (rectal/tympanic/forehead) are recommended for episodic measurement, abdominal skin temperature, despite it being subject to the vasomotor activity of the skin, is appropriate for continuous monitoring (figure 1A,B). In addition to being close to the liver, a metabolically active organ facilitating a measurement close to the core temperature,^10^ it is also a non-invasive method that is steady, continuous, easy-to-use and comfortable for the infant.^11^ The temperature ranges of normal and abnormal (hypothermia or hyperthermia) are shown in figure 2.

### Innovation pathway

We adopted the USAID 2006 Innovation Pathway model that incorporated four steps from research to field implementation: (1) priority-setting phase and product design, (2) product development and proof of concept, (3) product introduction and (4) field implementation.^12^ As part of step 1, we reviewed the substeps of (1) problem identification (epidemiological/technological/social/financial), (2) critical review of temperature measurement devices and other issues (temperature sensors or thermistors, core body temperature measurement issues) and (3) determination of niche of this device vis-à-vis other devices through horizon scanning.

Epidemiological burden of chief causes of maternal and neonatal mortality and morbidity were reviewed as a first step.^4 5^ A technological review revealed that the device to be strapped onto the mother or newborn for vital sign detection had to be a medical-grade device with extremely high safety profile. Further, it had to remain functional in situations such as soiling or wetting by the newborn, and also continue to work in different geographic areas with varying population densities as well as buildings. Several devices such as the ThermoSpot, a temperature sensor tag and remote infrared-based instruments were evaluated. Social review revealed that a sensor being continually strapped on to the body for long periods was not dissimilar to that of talismans strapped around the arms of adults or around the necks/waists of children and might therefore not be too disagreeable to families. A financial review revealed that for the device to be used widely, especially by low-income populations, it would have to be a low-cost device. Implementation review focused on possible uptake and utilisation of such a device in rural, urban and slum communities. The focus of this paper is to report on the clinical evaluation of the product as part of step 2 of the innovation pathway.

### Conceptual framework

**Figure 3:**
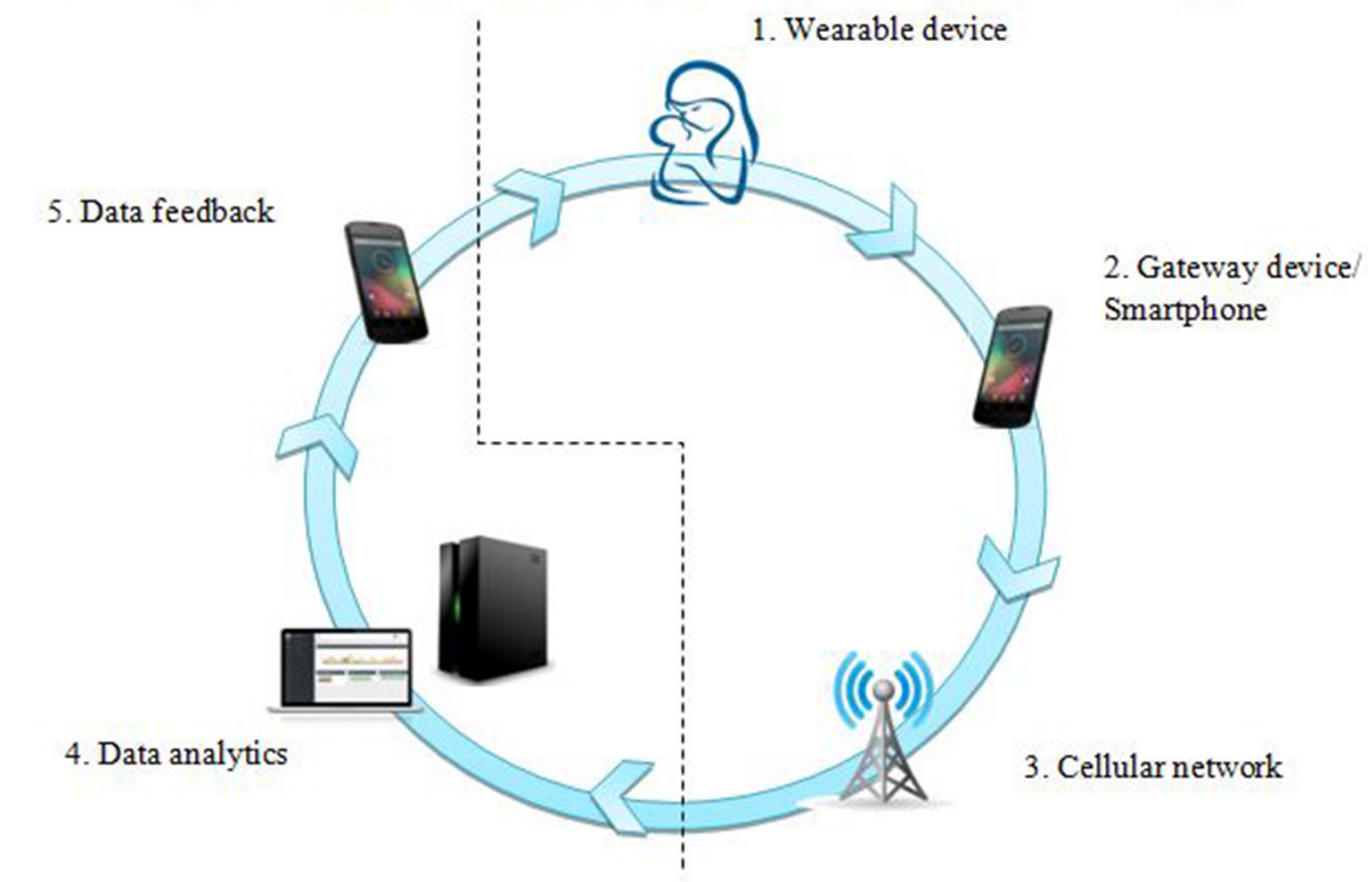
Conceptual framework for remote biomonitoring for temperature surveillance.

The conceptual framework for remote monitoring is illustrated in figure 3. The innovation framework consisted of five components: (1) a low-cost, wearable sensor tag; (2) a gateway device acting as an ultra-low-power ‘real-time’ communication link; (3) piggy-backing on a commercial cellular communication network; (4) smart data analytics system and (5) feedback loop to the caregiver or front-line healthworker. This framework was to be ideated, designed, prototyped and developed into a Class A medical device with lowest risk level as per Medical Device Regulatory Authority of India^13^ bearing in mind that the end product should enable tenets of good QoC, namely effectiveness, timeliness, safety, people-centredness and equity.^14^ Here we describe the functioning of the first two of five components.

### Requirements for components

Two key requirements for the ‘on-body’ sensor were safety and accuracy. Given the fragility of the newborn skin,^15^ the device had to be hypoallergenic,^16^ burst/leak proof,^17^ cause minimal infections and dissipate minimal heat or ultra-low-power, non-ionising electromagnetic radiation.^18^ The adhesive used to secure the device to the skin should similarly cause minimal ‘medical adhesive-related skin injury’,^19^ allergy or infections. Device accuracy was targeted to be ±0.2°C in *in vitro* conditions and ±0.5°C in actual clinical practice. Other mechanical requirements for the device were long battery life up to 28 days (with sampling frequency of 5 min); robustness (without any malfunction on coming into contact with body fluids like sweat, blood, urine, faeces; at least ‘ingress protection class 67’ (dustproof and waterproof); human-centric and aesthetic design for non-intrusiveness over prolonged use; and ability to withstand mild shock or vibration; and that the device should not get reset accidentally (online supplementary figure 1). There was also a requirement for the device to store data locally and communicate with a gateway device for onward transmission of data via the configured cellular network.

10.1136/bmjinnov-2016-000153.supp1 Supplementary file 1

### Product design and engineering

**Figure 4:**
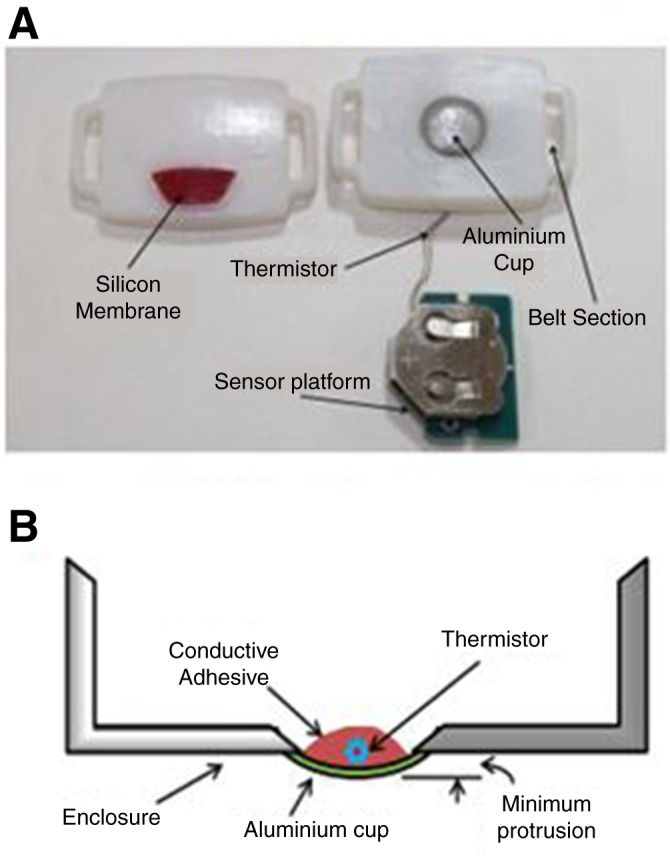
(A) Disassembled prototype; (B) vertical cross-sectional view of enclosure.

Based on the clinical requirements, a preliminary design was constituted and subsequent design choices underwent multiple iterations driven by technological capabilities and user reviews. The wearable sensor enclosures were pebble-shaped or coin-stack-shaped with all the electronics embedded inside (online supplementary figure 1; figure 4A,B). A battery of tests (both mechanical and electrical) were undertaken to confirm device performance, robustness and reliability. After several rounds of pre-testing, design optimisation was achieved.

### Prototype and implementation

The details of the prototype sensor device are given elsewhere.^20^ Briefly, the sensor hardware platform, developed using a multilayer printed circuit board, consisted of a microcontroller with integrated Bluetooth V.4.0 Low Energy, a 12-bit ADC (CC2540 from Texas Instruments), a NICU-grade temperature sensor with its analog front-end circuit, status LEDs, power supply and RF balun filter and antenna for wireless communication over 2.4 GHz ISM band. High-precision MF51E NTC thermistors (Cantherm, 2009)^i^ were used for extremely accurate temperature measurements. An embedded Inverted F-antenna with higher efficiency, longer range and a wider bandwidth than a chip antenna was used. It also had a very low-tolerance resistor enabling a low-power consumption during both active (150 μA) and sleep (1 μA) modes. The sensor hardware was programmable as per requirements. A 3 V coin battery powered the device. A baby-friendly enclosure was made from medical-grade hypoallergenic plastics (figure 4A,B; online supplementary figures 1 and 2).

The sensor communicated with a gateway device (a smartphone or a Raspberry pi) that could subsequently relay the temperature data over a secured internet backbone provided by GPRS/Wi-Fi onto a centralised database for storage (online supplementary figure 3).

### Phases of device testing

The planned phases of device testing were pre-clinical testing (in June 2013) and clinical trial phases I and IIa for the evaluation of safety and efficacy in the following sequence: healthy adult volunteers (May 2014); healthy mothers (January to February 2015); healthy babies (February 2015); stable babies in the NICU (February to March 2015) and babies with morbidities such as hypoxic ischaemic encephalopathy (HIE) (March 2015). The results of the pre-clinical testing in the laboratory setting were published earlier.^20^ Briefly, the response-time of the sensor device to attain thermal equilibrium with the surroundings was 4 min compared with 3 min observed with a precision-grade digital thermometer used as a reference standard. In terms of accuracy, the error was calculated to be within 0.1°C of the reference standard while using waterbaths in the temperature range of 25–40°C (online supplementary figure 4). The details of the clinical phase of testing are outlined below.

Seven free-living healthy adult volunteers (males=2; females=5), with no known morbidities, were the first-phase participants over a 7-day period. All of them had the devices strapped with an armband secured with Velcro tape onto their left upper arms (figure 1A) and were invited to contribute at least a minimum of 24 hours of observations accumulated from over one or more days and report any adverse events or side effects they experienced. In parallel, they also noted down timed axillary temperature readings (at least five times in a 24-hour window) using a digital thermometer for paired comparisons.

Testing in healthy postnatal mothers (n=11) was first carried out among those with well babies in the postnatal ward and then among mothers (n=7) with neonates admitted in the NICU. The devices were secured onto their upper arms with armband secured with Velcro tape. Paired readings taken every 5 min over a 1-hour period with an axillary digital thermometer were compared against the readings of the sensor with its time stamp.

Testing among neonates was carried out in three different phases in all of which the devices were secured with cotton and micropore adhesive to the upper epigastrium. In the first phase, well babies (n=3) in their early neonatal periods in the postnatal ward had their axillary temperature readings taken every 5 min over a 1-hour period with a digital thermometer and compared against the sensor readings. In the second phase, sick but stable neonates (n=10) from the NICU were recruited. They were under the radiant warmer with the temperature probe fixed onto the upper abdomen beside the sensor device and so the readings taken every 5 min over a 1-hour period from the warmer panel were compared against the sensor readings. But since all these babies had their temperatures maintained within a narrow normal range under the radiant warmer, we included one sick infant with HIE due to birth asphyxia and who was on treatment with therapeutic whole-body cooling. This facilitated comparison of readings in the range of 33–34°C during the cooling and rewarming phases of the treatment. The radiant warmer probe readings taken every 5 min over a 1-hour period from the warmer panel were compared against the sensor readings.

### Definitions

#### Safety

We instituted an adverse event reporting and resolution protocol for the wearable sensor devices. This enabled capture of the number and severity of adverse events as well as the individual clinical management as also feedback for changes into device design. Adverse events to be recorded were dermatitis, infection, thermal injury, radiation injury, device leak/burst and others.

#### Accuracy

Accuracy of the device was estimated by comparing temperatures recorded by the device against other measurements routinely used in clinical practice. For mothers and well babies in postnatal wards, the comparisons were between the device temperatures versus axillary temperatures read from a digital thermometer (once readings stabilised after the beep—usually after 3 min); for newborns in NICU, the comparisons were between the time-stamped device temperatures versus skin probe temperatures obtained from the control panels of calibrated radiant warmers (Phoenix Medical Systems Private Limited, Chennai, India, or Zeal Medical, Mumbai, India).

### Statistical analysis

Mean (±SD) was calculated for the paired sets of readings noted in mothers and newborns and the mean differences were obtained. Paired t-test was used for testing of significance between two different methods.

## Results

The seven adult volunteers, aged 20–45 years, contributed a total of 345 hours of readings (range=25–111 hours) over the 7-day period. No major adverse events were noted; four of seven participants noted a minor adverse event of sweating under the device/armband and one participant developed mild skin allergy with copper coating of the sensor.

The first set of 11 postnatal mothers provided a total of 312 paired readings. Ten of them had their temperatures measured during the mid-day (10:30 to 15:30) and one mother had her temperatures recorded in the early morning (between 5:00 and 6:30). The second set of seven postnatal mothers provided a total of 91 paired readings during the mid-day period (10:30 to 13:30). No adverse events were noted in any of the mothers. The mean skin temperature measured by the sensor was 2°C lower than the axillary temperature readings (sensor=34.1°C vs digital=36.1°C) in the volunteers as well as both sets of mothers and this difference was statistically significant (t-test=13.8; P<0.001) (table 1) (online supplementary figure 5).

**Table 1:** Results of remote biomonitoring device testing of upper arm skin temperature measurement against axillary digital thermometer measurement in postnatal mothers

Measurements: Postnatal mothers’ categories	Time of temperature recording (hh:mm)		Readings (n)	Temperature Mean (±SD)		Temperature Mean difference
	(first reading)	(last reading)		Device (upper arm)	Digital (axilla)	(device-digital)
Mothers of well babies						
1	11:17	12:17	13	35.34	36.8	−1.4
2	11:02	12:02	13	35.15	36.7	−1.5
3	11:03	12:03	13	33.69	36.0	−2.3
4	11:18	12:18	13	34.02	36.4	−2.4
5	10:36	11:36	13	33.18	36.1	−2.9
6	11:50	12:50	13	34.21	36.2	−1.9
7	11:31	12:31	13	33.75	35.8	−2.1
8	11:38	12:38	13	33.31	36.1	−2.8
9	12:32	13:32	13	33.88	35.7	−1.8
10	14:19	15:19	13	35.69	36.4	−0.7
11	05:08	06:18	4	33.00	35.5	−2.5
Subgroup	–	–	312	34.1 (±0.9)	36.1 (±0.4)	−2.0*
Mothers of sick neonates						
12	12:27	13:27	13	32.85	35.3	−2.4
13	11:08	12:08	13	35.29	36.5	−1.2
14	11:30	12:30	13	33.35	36.4	−3.1
15	14:18	15:18	13	34.16	36.4	−2.2
16	10:25	11:25	13	33.39	35.9	−2.5
17	11:41	12:41	13	35.16	36.4	−1.3
18	11:16	12:16	13	33.97	36.1	−2.2
Subgroup	–	–	91	34.0 (±0.9)	36.1 (±0.4)	−2.1†
Overall	–	–	403	34.1 (±0.9)	36.1 (±0.4)	−2.0‡

All  P values < 0.001.

*t-test=10.5; df=10.

†t-test=8.3; df=6.

‡t-test=13.8; df=17.

The first set of three neonates provided a total of 39 paired readings during the early morning time window (4:00 to 5:00). The mean difference in temperature was 0.14°C (sensor=36.87°C vs digital=36.73°C; P=0.2). The second set of 10 neonates in the NICU provided a total of 130 paired readings. Their mean skin temperature measured by the sensor was 0.6°C lower than that measured by the radiant warmer probe (sensor=35.88°C vs warmer probe=36.46°C; P<0.001). The last phase study neonate in the NICU provided a total of 25 paired readings with the mean sensor reading being not different from the radian warmer probe reading (sensor=33.53°C vs warmer probe=33.54°C; P=0.8) (table 2). No adverse events were noted in any of the babies.

**Table 2:** Results of remote biomonitoring device testing of newborn baby temperature measurement in different temperature ranges by different groups of neonates

Measurements: Neonates’ categories	Time of temperature recording (hh:mm)		Readings (n)	Temperature Mean (±SD)		Temperature Mean difference
	(first reading)	(last reading)		Device (abdomen)	Digital (axilla)	(device-digital)
Well babies (in postnatal ward)						
1	03:54	04: 54	13	36.61	36.6	0.01
2	04:02	05: 02	13	37.00	36.8	0.18
3	04:05	05: 05	13	37.00	36.8	0.21
Subgroup	–	–	39	36.87 (±0.2)	36.73 (±0.1)	0.13*
				Device (abdomen)	Probe (abdomen)	(device-probe)
Sick neonates in NICU under radiant warmer with temperature >35.5°C						
4	15:43	16:43	13	36.62	36.57	0.05
5	11:16	12:16	13	35.78	36.56	−0.78
6	12:47	13:47	13	35.66	36.34	−0.68
7	11:56	12:56	13	35.61	36.64	−1.03
8	14:26	15:26	13	35.50	36.35	−0.85
9	11:25	12:25	13	35.41	36.32	−0.91
10	14:00	15:00	13	35.76	36.43	−0.67
11	11:04	12:04	13	36.71	36.45	0.26
12	12:17	13:17	13	35.84	36.43	−0.59
13	12:39	13:39	13	35.89	36.47	−0.58
Subgroup	–	–	130	35.88 (±0.4)	36.46 (±0.1)	−0.6†
Sick neonate in NICU						
Undergoing therapeutic cooling for birth asphyxia (temperature approximately 33°C)						
Neonate 14	02:51	03:51	13	33.10	33.15	0.05
Undergoing rewarming after therapeutic cooling for birth asphyxia (temperature approximately 34°C)						
Neonate 14			12	33.95	33.92	0.03
Subgroup	–	–	25	33.53 (±-0.6)	33.54 (±0.5)	0.01‡
Overall	–	–	155	35.76 (±1.1)	36.12 (±1.1)	−0.4§

*t=2.2; df=2; P=0.2.

†t=4.4; df=9; P<0.001.

‡t=0.25; df=1; P=0.8.

§t=3.0; df=14; P<0.01.

NICU, neonatal intensive **care** unit.

## Discussion

Our RBM device, a battery-operated, wearable temperature sensor tag designed and developed based on two sets of criteria, namely client needs and technological requirements, was within an enclosure package that took into account design elements and incorporated hermetic sealing with no sharp corners/crevices for accumulation of bacteria/dust and designed to be easily sterilisable. It is a product built on a ‘needs-based biodesign innovation’^21^ and qualifies as a breakthrough product that could potentially offer a clinically meaningful advantage for life-threatening conditions in resource-poor settings where no alternate means of diagnosis exists for mothers and newborns. The biomonitoring sensor satisfies the definition of an innovation with characteristics of novelty and application with a clearly intended benefit.^22^

In our study, skin temperatures in mothers were lower than the axillary temperatures by 2°C; this expected difference can be used to calibrate the device readings in future when used for temperature surveillance in adults. In newborns, it had a precision of ±0.5°C relative to axillary measurements at 15 min intervals and was comfortable to wear. Our innovation was similar to other products such as the unobtrusive continuous temperature monitoring of infants in NICUs reported elsewhere.^10^ Differences in the reporting of sweating under the device/band by adult volunteers and mothers were possibly due to differences in environmental temperatures during different testing seasons. Allergy reports led to design modifications with a gold/stainless steel plating over the sensor surface.

Our current round of testing is not without limitations. Our preliminary round of testing has revealed the health technology to be safe (in the short term) and accurate for short duration testing. The next step would be to test the sensor and other technologies for longer durations (days to weeks) in both mothers and newborns. Further, conversion of the sensor into a multiparameter detection device is likely to render it more useful in clinical and public health settings as also improving the cost-effectiveness of the device. Another major limitation of our current approach was the use of trial-and-error methods in testing which resulted in cost and time over-runs. In the next rounds of testing, more rigorous approaches may be employed across the phases of design, development and testing.^23 24^ Though our device has been found to be safe in the short term, additional in vitro and in vivo safety testing must continue on the long-term safety of early neonatal exposure to contact devices as well as low-dose radiofrequency radiation.^25^

The sensor device will also have to be tested for its ability to not only ‘sense’ data on neonatal temperatures but also to act as a feedback tool for thermoprotective measures. It could also be used to collect information on possible risk factors such as maternal temperature, environmental conditions and sociocultural practices and then based on back-end analytics using predefined algorithms, specific messages could be sent to hospitals, families or community health workers for preventive/corrective measures including behaviour change or referral of sick newborns (figures 2 and 3 and online supplementary figure 3).^6^

In summary, this proof-of-concept study shows this early-stage innovation^26^ to be promising in terms of safety and accuracy (with appropriate calibration) for monitoring of temperatures in adults and newborns that can potentially be carried forward to the next stages of device accuracy refinement as well as further phases of clinical research for improved maternal and neonatal health. Investing in such innovations would be critical to achieving maternal and neonatal health goals over the next decade.^1 6 27^

## Footnotes

i Cantherm (2009). High precision NTC thermistors. http://www.cantherm.com/products/thermistors/mf51e.html/webfigures_20160812.pdf.
