# Supplementary material for: Remote biomonitoring of temperatures in mothers and newborns: design, development and testing of a wearable sensor device in a tertiary-care hospital in southern India
Source: BMJ Innov. 2018 Feb 14;4(2):60–7. doi: 10.1136/bmjinnov-2016-000153 (PMC5890619; doi:10.1136/bmjinnov-2016-000153)

Web appendix 1

Web figure 1. Baby-friendly enclosure

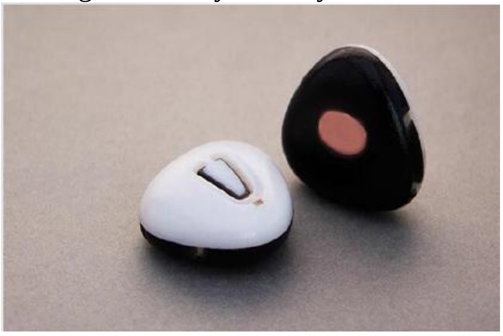

Web figure 2. Top and bottom view of the sensor

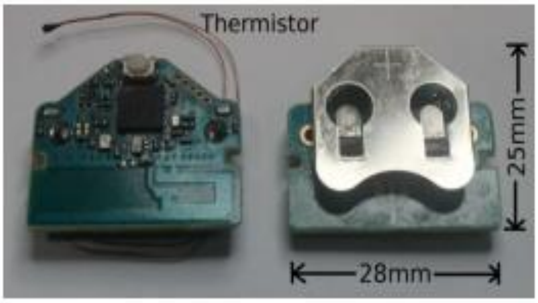

Web figure 3. Temperature readings on the cellphone through wireless capability

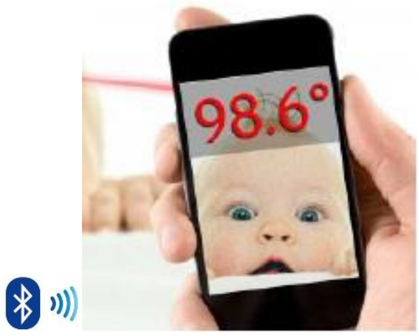

Web figure 4. Responsiveness and accuracy measurement of the sensor

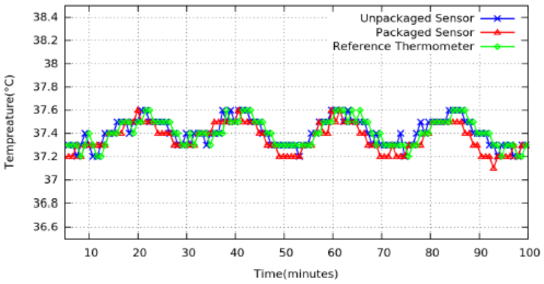

(a) Temp. measurements with hot plate at 37°C

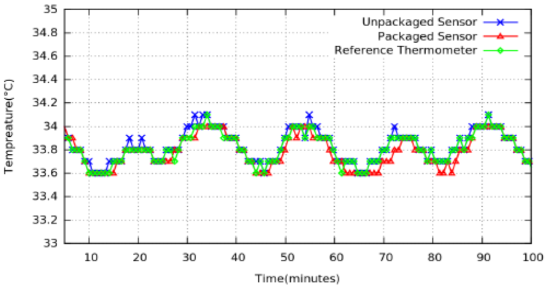

(b) Temp. measurements with hot plate at 34°C

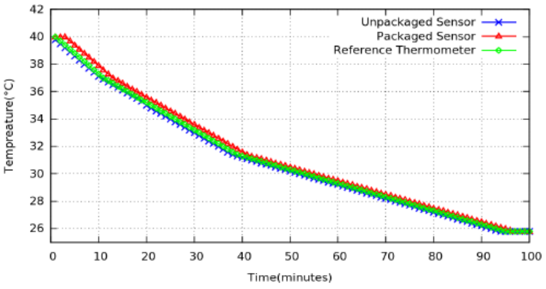

(c) Temperature measurements without hot plate

Web figure 5. Temperature graphs constructed as part of exploratory data analysis for (a) human volunteers and (b) postnatal mothers

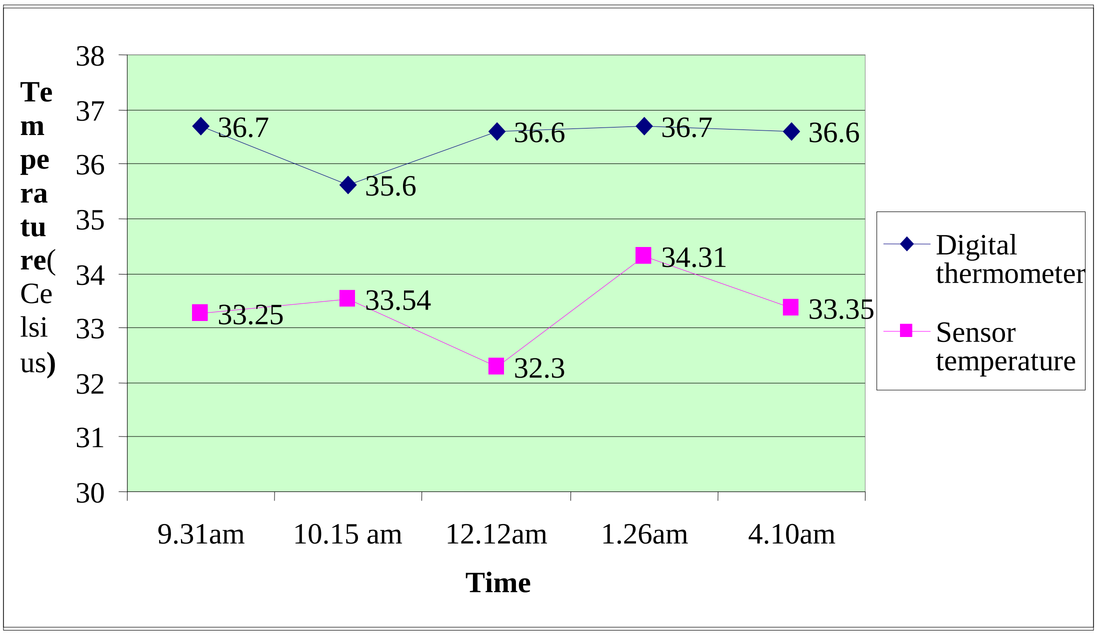

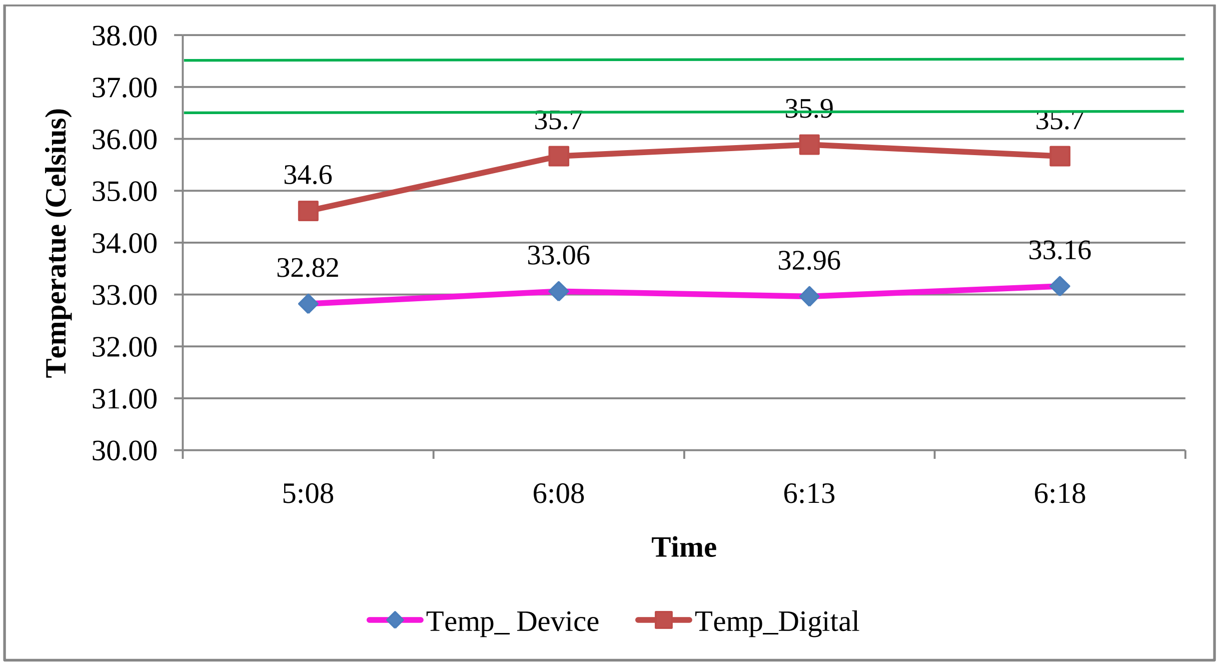

Supplement: Supplementary file 1 [file bmjinnov-2016-000153supp001.pdf]
